# Effects of Type 1 Diabetes Risk Alleles on Immune Cell Gene Expression

**DOI:** 10.3390/genes8060167

**Published:** 2017-06-21

**Authors:** Ramesh Ram, Grant Morahan

**Affiliations:** 1 Centre for Diabetes Research, Harry Perkins Institute of Medical Research, Nedlands, WA 6009, Australia; ramesh.ram@uwa.edu.au; 2 Centre of Medical Research, University of Western Australia, Nedlands, WA 6009, Australia

**Keywords:** type 1 diabetes, eQTLs, B-cells, T-cells, dendritic cells

## Abstract

Genetic studies have identified 61 variants associated with the risk of developing Type 1 Diabetes (T1D). The functions of most of the non-HLA (Human Leukocyte Antigen) genetic variants remain unknown. We found that only 16 of these risk variants could potentially be linked to a protein-coding change. Therefore, we investigated whether these variants affected susceptibility by regulating changes in gene expression. To do so, we examined whole transcriptome profiles of 600 samples from the Type 1 Diabetes Genetics Consortium (T1DGC). These comprised four different immune cell types (Epstein-Barr virus (EBV)-transformed B cells, either basal or after stimulation; and cluster of differentiation (CD)4+ and CD8+ T cells). Many of the T1D-associated risk variants regulated expression of either neighboring (*cis*-) or distant (*trans*-) genes. In brief, 24 of the non-HLA T1D variants affected the expression of 31 nearby genes (*cis*) while 25 affected 38 distant genes (*trans*). The effects were highly significant (False Discovery Rate *p* < 0.001). In addition, we searched in public databases for expression effects of T1D single nucleotide polymorphisms (SNPs) in other immune cell types such as CD14+ monocytes, lipopolysaccharide (LPS) stimulated monocytes, and CD19+ B cells. In this paper, we review the (expression quantitative trait loci (eQTLs) associated with each of the 60 T1D variants and provide a summary of the genes impacted by T1D risk alleles in various immune cells. We then review the methodological steps involved in analyzing the function of genome wide association studies (GWAS)-identified variants, with emphasis on those affecting gene expression. We also discuss recent advancements in the methodologies and their advantages. We conclude by suggesting future study designs that will aid in the study of T1D risk variants.

## 1. Introduction

**Figure 1:**
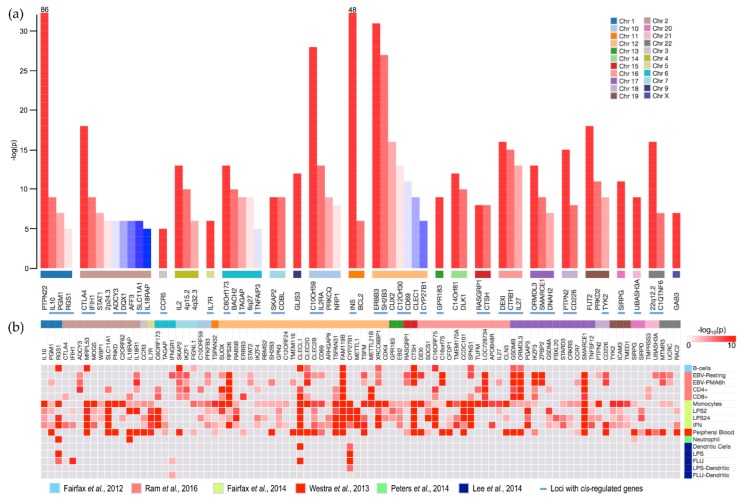
Summary of Type 1 Diabetes (T1D) loci and candidate *cis*-regulated genes in immune cells: (**a**) Non-HLA (human leukocyte antigen) T1D risk genes along with reported genome wide association studies (GWAS) association *p*-values; (**b**) *Cis*-regulated genes that were validated in more than one cell type. The grey cells indicate no significant regulation found. Shades of red represent the negative log_10_(*P*) association. The loci and *cis*-regulated genes are grouped according to chromosome. All loci with *cis*-regulated genes are highlighted by blue dashes.

Four [1,2,3,4] genome wide association studies (GWAS), a linkage study [5], and five [6,7,8,9,10] other studies have identified 61 genetic variants that confer risk of Type 1 Diabetes (T1D). In Figure 1a, we summarize 60 non-HLA (Human Leukocyte Antigen) T1D risk loci identified so far. GWAS typically involves (a) recruiting patient cohorts accurately diagnosed with the disease; (b) recruitment of healthy controls; (c) sample collection and genotyping of individuals, typically at 500,000 markers (Single Nucleotide Polymorphisms (SNPs)); (d) Imputation of potentially millions more SNPs; and (e) performing association analysis of each SNP with the disease or trait of interest. All SNPs that exceed stringent thresholds of significance (*p* < 5 × 10^−8^, suggestive *p* < 1 × 10^−5^) are deemed to be associated with the disease. It is understood that known covariates (e.g., age, sex) and unknown confounders (e.g., relatedness) could limit gene discovery, however, statistical approaches such as principal component analysis (PCA) [11], clustering analysis [12], and linear mixed models [13] have helped overcome such problems. Validation cohorts and studies in different populations provide confirmation and improve confidence of gene-disease associations. However, all SNPs identified above genomic significance thresholds are treated as important. The results of GWAS are commonly summarized as Manhattan plots where the X-axis is the genomic coordinates and the Y-axis is the negative logarithm of the associated *p*-value for each SNP. Among the SNPs that exceed the significance threshold, each risk locus is defined by a lead SNP with maximum association signal (lowest *p*-value) and all SNPs in ±0.5 Mb of the lead SNP are excluded. Testing the genes nearest to the lead SNPs potentially reveals a link between the locus and the disease, even though this may not be the best method for establishing candidacy due to lack of evidence linking the lead SNP to the target gene. Nevertheless, in GWAS of T1D, lead SNPs were found adjacent to genes like *HLA* [14], *PTPN22* [15], *INS* [16], *CTLA4* [17], and *IL2RA* (also known as *CD25*) [18] that have all been reported since the pre-GWAS era.

The T1D risk loci are interconnected (directly or indirectly) via networks; perturbations of these networks presumably cause disease. This could arise from the inability (or decreased ability) of the products of disease genes to interact with other gene products when such interactions are required. This may be caused by factors including: (a) functional variants; (b) gene expression changes; and (c) epigenetic aberrations such as differential methylation. Regarding effects on the protein sequence of the gene, amino acid changes caused by missense SNPs could either be tolerated or damaging; in both cases the protein’s function could be impacted. Similarly, the risk SNPs may affect transcription rates of genes.

To formally establish disease gene candidacy, we performed a study [19] systematically evaluating each of 60 T1D risk loci. This revealed genes affected by missense SNPs and genes whose expression levels were perturbed with respect to T1D SNPs. We found that only 16 of the risk variants could potentially be linked to a protein-coding change. Expression effects were tested using 600 samples from the Type 1 Diabetes Genetics Consortium (T1DGC) comprising four cell types (a) EBV (Epstein-Barr virus) resting cells (b) PMA (Phorbol 12-myristate 13-acetate) stimulated EBV cells (c) cluster of differentiation (CD)4+ T-cells, and (d) CD8+ T-cells. In these analyses, each T1D SNP was tested against the whole transcriptome as individual traits and the association *p*-values were derived. The genes were categorized based on their proximity to the corresponding T1D SNPs as: (a) *cis*-regulated genes i.e., those within ±1 MB of the SNP and the remaining (b) *trans*-regulated genes. The false discovery rate (FDR) method [20] was employed to allow for multiple testing; genes with *FDR* < 0.05 were deemed significant. The aims of this analysis were to identify genes that were up or down regulated according to the risk SNP genotypes and to identify pathways and networks affected. Similar expression studies [21,22,23,24,25,26] have analyzed the effect of SNPs (inclusive of T1D SNPs) on expression of genes in eight cell types: (a) monocytes; (b) interferon-*γ* (IFN) stimulated monocytes; (c) lipopolysaccharide (LPS) stimulated monocytes; (d) B-cells; (e) neutrophils; (f) dendritic cells; (g) LPSs; and (h) FLU (Influenza virus) infected dendritic cells. A large meta-analysis of gene expression effects in peripheral blood was also presented [25]. The associations in peripheral blood serve as validation for associations found in other cell types. Individually, these studies had reported adjusted (FDR) and unadjusted *p*-values for the SNP-gene associations that were deemed significant by them using suitable FDR thresholds. We searched in these databases for gene expression effects of only T1D SNPs. Based on the evidence available, we compared *cis*- and *trans*- regulated genes of each one of the T1D SNPs in various immune cells and peripheral blood.

The results are summarized as genes regulated in multiple cells and cell-type specific genes. FDR corrections remain specific to each study and expression quantitative trait loci (eQTLs) deemed significant in each study were retrieved from their corresponding supplementary tables. In these supplementary tables, we looked for reported eQTLs involving either lead T1D SNPs or any SNPs in high linkage disequilibrium (LD) (i.e., *r*^2^ > 0.8) with the lead T1D SNPs. The list of LD SNPs was obtained using LDlink [27]. Genes whose expression is associated with T1D SNPs in more than one cell type are considered validated. However, for cell-type specific genes, only eQTLs reported below arbitrary *p*-value thresholds of *p* < 1 × 10^−4^ (*cis*-regulated) and *p* < 1 × 10^−8^ (*trans*-regulated) were investigated due to their lack of validation in other cells; the excluded eQTLs reported outside this P range are nevertheless significant (as per the study-specific FDR thresholds). The lack of overlap of these eQTLs in multiple cells can always be due to lack of power, however, cell-specificity cannot be ruled out. Since genes in HLA have been studied elsewhere, the function of most of the non-HLA genetic variants remains unknown, thus, we focus only on the non-HLA T1D SNPs. Enrichment analysis was performed using the Molecular Signature Database (MsigDB) [28] to describe the possible biological mechanism of the identified *cis*- and *trans*- regulated genes.

## 2. Gene Expression Studies in Immune Cells

The T1D risk SNPs are summarized in Figure 1a and reviewed in (refer to Table 1 in ref. [5,19]). There has also been a flurry of publications [19,21,22,23,24,25,26,29,30,31,32,33] investigating the role of risk SNPs regulating gene expression. Of these, seven studies [19,21,22,23,24,25,26] focused on cells involved in the immune system and autoimmune diseases, hence, they are reviewed here. Firstly, we performed expression analysis in four immune cells [19]: EBV transformed lymphoblastoid cell lines (LCLs) (resting and PMA stimulated) and CD4+ and CD8+ T-cells of 600 subjects from the Type 1 Diabetes Genetics Consortium cohort (T1DGC). The cohort comprised families of affected siblings. We identified 76 genes *cis*-regulated by T1D SNPs with FDR < 0.05 (min *p* < 0.0008). Thirteen genes were *cis*-regulated in CD4+ and CD8+ cells, while 11 were *cis*-regulated in EBV and T-cells. In addition, we also identified 37 *trans*-regulated genes (min *p* < 1 × 10^−8^). Secondly, Fairfax and colleagues reported the expression effects in CD19+ B-cells and CD14+ monocytes from 239 healthy volunteers of European ancestry [21]. Among the list of significant associations reported, in the context of T1D SNPs there were 18 *cis*-regulated genes and 3 *trans*-regulated genes. Subsequently, they also reported expression analysis in CD19+ Monocytes in 432 healthy volunteers in [22] where monocytes were exposed to either interferon-*γ* or lipopolysaccharide (LPS) for a short (2-h) or long (24-h) duration. The study concluded that the majority of the *cis* genes identified were condition-specific. Among these results, some 90 genes were *cis*-regulated in the context of T1D SNPs and only five genes were *trans*-regulated. A third study [23] reported expression effects in dendritic cells isolated from the peripheral blood monocytes (PBMCs) of 560 healthy individuals of mixed ancestry (European, Asian, or African-American) from the PhenoGenetic cohort. Dendritic cells were stimulated with LPS, influenza virus, or interferon-*β*. Among the 385 *cis*-eQTLs identified in this study, seven were associated with T1D SNPs. Fourthly, Peters et al. [24] reported eQTL effects in primary immune cells (CD14+ monocytes, CD19+ B-cells, CD4+, and CD8+ T-cells) in 180 subjects. A novel joint analysis found *cis*-eQTLs that were cell-type and condition-specific. Twenty of the *cis*-regulated genes reported in [19] were associated with T1D SNPs. Lastly, Westra et al. [25,26] performed a large scale meta-analysis of expression eQTLs in peripheral blood and reported *cis*- and *trans*- genetic associations using a less stringent threshold of FDR < 0.5, finding 77 *cis*- and 127 *trans*-regulated genes in association with T1D SNPs. We combined the results from the above studies such that each record would contain: SNP identifier (rsID), T1D locus name, gene name, unadjusted *p*-value, cell-type, and publication reference. These data are summarized in Supplementary Materials Table S1.

## 3. Candidate Gene Identification

This section is sub-divided into *cis*-regulated genes and *trans*-regulated genes.

### 3.1. Cis-Regulated Genes

Most studies considered *cis*-regulated genes as being located within the ±1 MB of the SNP position. Our search for genetic interaction associated with T1D SNPs in seven cell types [19,21,22,23,24,25,26] revealed 90 genes that showed significant (FDR < 0.05) association in more than one cell type and 34 genes that were cell type-specific. In Figure 1b, we show the 90 genes and their association with 40 T1D loci where unadjusted association *p*-values extracted from corresponding studies are indicated as a heatmap. These associations may be considered validated as they were replicated in multiple cell types. Interestingly, eleven of the 90 genes were located in chromosome band 12q13 (commonly referred to as the ERBB3 locus [34]). Additionally, cytogenetic bands 17q21 (*ORMDL3*) and 16p11 (*IL27*) included six and five *cis*-regulated genes, respectively. The remaining risk loci were associated with expression of fewer genes. Enrichment analyses were performed in the Canonical pathways of KEGG (www.genome.jp/kegg/) and Reactome (www.reactome.org) databases. In doing so, eight genes (*IFNGR1*, *IL7R*, *IL18R1*, *CCR3*, *CCR7*, *IL10*, *IL18RAP*, and *TNFSF12*) were associated with the cytokine-cytokine receptor interaction pathway (KEGG) (*p* = 3.82 × 10^−8^) while 12 genes (*IFNGR1*, *IL7R*, *SOCS1*, *TYK2*, *STAT2*, *PTPN2*, *CTLA4*, *RASGRP1*, *CTSH*, *ICAM3*, *CD226*, and *IFIH1*) were associated with immune system (Reactome) pathways (*p* = 1.55 × 10^−7^). The remaining genes were tested for enrichment in the Gene Ontology (GO) database. An additional 11 genes (*GPR183*, *IKZF3*, *RAC2*, *TSPAN32*, *SKAP2*, *IL27*, *SIRPG*, *SLC11A1*, *UBASH3A*, *CLEC2D*, and *CLEC2B*) were associated with GO regulation of “immune system process” (GO:0002682) (*p* = 4.02 × 10^−18^). In addition to immune system or response terms, significant (FDR < 0.05) enrichment was observed for “cell activation”, “cell proliferation”, “leucocyte/lymphocyte activation”, and “defense response”.

**Figure 2:**
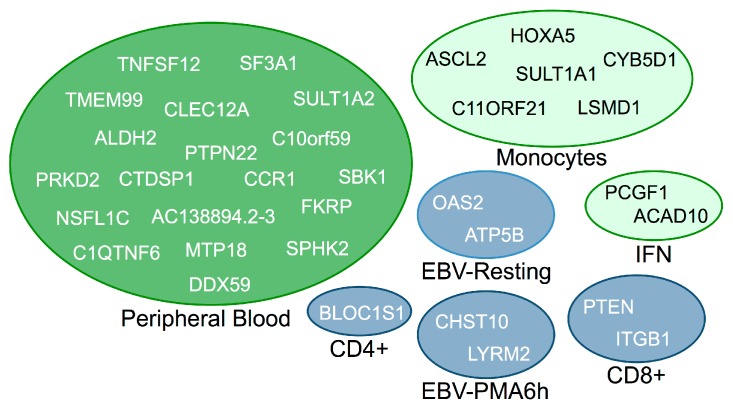
*Cis*-regulated genes that are cell-type specific. IFN denotes interferon-*γ* stimulated monocyte cells.

Only *cis*-genes reported with high confidence (*p* < 1 × 10^−4^) were selected for further analysis, irrespective of their reported FDR *p*-values. There were 34 such genes, the majority of which showed association only in peripheral blood cells. Cell-type specific *cis*-regulated genes are summarized in Figure 2.

Upon combining the list of cell-specific genes with ubiquitously expressed genes (*n* = 124 genes), there was an increase in the number of *cis*-regulated genes (from 3 to 7) associated with the 12q24 (*SH2B3*) locus. Enrichment analyses showed 40 of the 124 *cis*-genes were related to the immune system. Among the cell-type specific genes, *OAS2* (EBV-resting), *PTEN*, and *ITGB1* (CD8+) were involved in immune system pathways (Reactome) (*p* = 1.51 × 10^−8^). The genes *IFNGR1*, *SOCS1,* and *PTPN2* affected regulation of the interferon gamma (*IFNG*) signaling pathway (Reactome). We performed further separate analysis of non-immune related genes. In doing so, we found that *SULTA1*, *SULTA2*, and *SUOX* affected the Sulphur metabolism pathway (KEGG) (*p* = 4.4 × 10^−6^). In a study [35] into a possible bacterial role in developing T1D, sequencing of fecal samples showed a higher number of reads mapping to sulfur metabolism in T1D cases compared to that of controls, suggesting interaction of microbiome and sulphur metabolism genes.

Additionally, twelve of the genes (*SULTA1*, *SULTA2*, *ATP5B*, *CLN3*, *SPHK2*, *PGAP3*, *MTMR3*, *ERBB3*, *STARD3*, *APOBR*, *ACAD10*, and *ORMDL3*) were also associated with lipid metabolic processes (*p* = 4.37 × 10^−7^). It was shown that people with T1D also displayed disturbances in plasma lipids such as hypertriglyceridemia and low high-density lipoprotein (HDL) cholesterol [36]. These analyses suggest a novel role of non-immune related genes mediating T1D.

### 3.2. Trans-Regulated Genes

Genes whose expression varied significantly in association with T1D SNP alleles, but were located more than 1 Mb from the SNP may be considered as *trans*-regulated genes. First, we searched for *trans*-regulated genes that are validated by their observations in multiple cell types (or studies) with FDR < 0.05. In searching for cell specific *trans*-regulated genes, we used a stringent threshold of *p* < 1 × 10^−8^ to deem the results as significant. There were ten such genes and their negative log(*P*) values of the associations in different cells are summarized in Table 1.

Interestingly, the insulin locus SNP showed significant association with *trans*-regulated gene aquaporin-9 (*AQP9*) in both CD4 and CD8 positive cells. *AQP9* is down regulated by insulin in obese type 2 diabetes mellitus (T2DM) patients [37]. There were four genes and two micro-RNAs that were associated with the *ERBB3* (12q13.2) T1D locus. In particular, *IP6K2* (Inositol hexakisphosphate kinase-2) showed consistent association with 12q13.2 in all cells except peripheral blood. *IP6K2* is abundantly present in pancreatic beta cells and may be involved in regulation of insulin exocytosis. Novel genes *LAP3P2*, a Leucine Aminopeptidase 3, and *MAFG-AS1*, a *MAF* gene, showed association in multiple cell types. *MAF* genes such as *MAFA* are known to promote pancreatic development and insulin transcription. The *trans*-regulation of *DPF2*, a zinc finger gene in CD4+ T-cells, was confirmed in peripheral blood. *UBE2L6*, a ubiquitin gene, and *STAT1* were *trans*-regulated by the same T1D SNPs located in the 12q24 locus, as also confirmed in peripheral blood [26]. Another ubiquitin gene, *UBASH3A*, is a well-known candidate gene for the 21q22.3 T1D locus [1]. In [38], it was found that the ubiquitin system could be associated with insulin signaling and might be affected in diabetes.

In cell-type specific *trans*-regulated genes, we identified 12 genes in EBV-B cells, seven genes in peripheral blood, two genes in CD4+ cells, and one gene in monocytes. The list of these genes is provided in Table 2, sorted according to association *p*-value along with the regulating T1D SNP. Enrichment analysis of *trans*-regulated genes alone showed three of the genes (*IRF8*, *ID2*, and *CCL5*) were validated targets of *c-MYC* transcriptional repression. In doing Gene Ontology enrichment, we found that ten of the *trans*-regulated genes were associated with “positive regulation of biosynthetic process”.

## 4. Alternative Methods for Studying eQTLs Associated with Disease SNPs

Recent studies [19,21,22,23,24,25,26] indicate that T1D-associated SNPs are likely to be eQTLs. Associations at T1D risk loci were consistent with eQTLs (both *cis* and *trans*) in three relevant immune-cell populations studied [19]: EBV—lymphoblastoid cell lines (LCLs) (unstimulated and stimulated) and CD4+ and CD8+ T-cells. In this review, we performed a comparison of the eQTLs in additional cells such as CD14+ monocytes (unstimulated and LPS- and IFN-gamma stimulated) [21,22], CD19+ B-cells [21], fluorescence-activated cell sorting (FACS)-purified neutrophils [23], and dendritic cells (unstimulated and stimulated) [24] to carefully examine the expression effects of T1D risk alleles on immune gene expression. The eQTL results were also compared against results reported in whole blood [25,26]. For each T1D locus, variants in high LD (*r*^2^ > 0.8) with any of the lead T1D variants were also examined for overlap with eQTLs. The authors of each of these studies employed suitable thresholds to determine true-positive *cis* and *trans*-eQTLs. The analysis we performed was a simple one-to-one comparison of the eQTL association results (assuming they are true) between the cell-types aimed at compiling a summary of validated target genes by virtue of their co-occurrence in more than one cell-type or study. The eQTLs that could not be validated in multiple cells were assumed to be cell-specific. The techniques used in this analysis have advantages and disadvantages. In this section we explore the techniques by providing step-by-step methods where we discuss some alternative approaches that have, at times, better advantages for deducing eQTL associations with disease associated variants.

### 4.1. Prediction of Functionality of Disease Associated Variants

The first step in the analysis of disease associated SNPs is to test whether or not the SNPs have an impact on the function of the proteins encoded by the relevant genes [19,39]. This is commonly achieved by means of prediction algorithms that determine whether or not a variant is deleterious or benign by means of a score. There are two types of predictions available: protein-based and more recently nucleotide-based. For several years, protein-based sorting intolerant from tolerant (SIFT) [40] and Polymorphism Phenotyping 2 (PolyPhen-2) [41] were the only prediction methods which were applied to non-synonymous coding region SNPs to identify whether or not the amino acid changes were deleterious.

More recently, with advancements such as Encyclopedia of DNA Elements (ENCODE) [42] and Functional Annotation of Mouse 5 (FANTOM5) [43], tools such as Combined Annotation–Dependent Depletion (CADD) [44], Deleterious Annotation of genetic variants (DANN) [45], Functional Analysis Through Hidden Markov Models (FATHMM) [46], and Linear Inference of Natural Selection from Interspersed Genomically coHerent elemenTs (LINSIGHT) [47] have been developed to predict functionality of variants outside the coding regions. These tools are based on supervised machine learning algorithms using a well-characterized training dataset. The prediction is usually given as a score, typically between 0 and 1, where scores >0.8 usually can be treated as functional. PredictSNP [48] and PredictSNP2 [49] are convenient web-based meta-predictors incorporating all available methods in arriving at a consensus prediction where a required variant can be searched using the corresponding rsIDs. Using these tools, it is possible to determine whether the identified lead GWAS SNP can be characterized prior to performing eQTL association testing.

### 4.2. Gene Expression Quantification

In earlier eQTL studies [19,20,21,22,23], whole-transcriptome profiling was performed on Illumina’s HT-12v4 bead arrays. Briefly, the processing of the expression data involved quantile or Robust Spline (RSN) normalization [50], quality control, and filtering. Probes with a detection *p*-value of <0.01 (Illumina GenomeStudio Software, Illumina, San Diego, CA, USA) in at least 5% of the samples were retained for further eQTL analysis. Popularly, software such as ReMOAT [51] (Re-annotation and Mapping of Oligonucleotide Array Technologies) were used to assess probe quality and probes considered as “bad” are removed. Alternatively, probe sequences were tested for unique alignment to the transcriptome as described in [19] for consideration for eQTL association testing, and probes whose sequences contained SNPs were filtered out. These stringent probe-mapping strategies were employed to filter out false-positives due to primer-polymorphisms and cross-hybridizations. In the case of latter studies such as [24,25,26] using RNA-seq for transcriptome profiling, the processing steps differ. Briefly a (variance stability transformation) VST-normalization is applied to the read counts obtained from the mapped RNA-seq data, which is then regularized log (rlog) transformed using R package DESeq2 [52] prior to further analysis.

### 4.3. Batch Effect Correction and Removing Unwanted Variations

In both RNA-seq and microarrays, it is commonly known that batch effects and their influence on normalization can result in spurious findings. Approaches (e.g., ComBat method [53]) have been proposed to remove unwanted variation caused by differences between batches of samples. Principal variation component analysis (PVCA) [54] has also been used to detect and correct batch effects where the Principal Components (PCs) attributed to batching can be subtracted by regression and residual expression calculated for further analysis. Some additional methods have been proposed based on the use of spike-in negative control probes such as SQN (subset quantile normalization) [55] and RUV (remove unwanted variation) [56] to remove other unknown variations that limit the rate of eQTL detection. Furthermore, unknown hidden variables can be also be detected by surrogate variable analysis (SVA) as described by Leek et al. [57]. SVA method is implemented in the R package “sva” [58]. Sample label mix-ups are another common problem that can often cause reduction in power to detect eQTLs. In [59], the authors introduce a method “MixupMapper” to correct such errors by comparing actual versus predicted gene expression for genes with very strong *cis*-eQTLs where the expressions are predicted solely based on SNPs.

### 4.4. Identify and Remove Known and Hidden Confounding Factors in the Normalized Expression Data

It is common practice to remove confounding factors from expression data as this limits the detection of true-positive eQTLs. To remove the effects of covariates such as age, sex, and HLA types, the R package “pedigreemm” [60] performs a mixed-effect modeling, accounting for relatedness between samples. This also allows for the calculation of the residual gene expression. To adjust for hidden factors, the residuals can be subjected to a Bayesian framework known as Probabilistic Estimation of Expression Residuals (PEER) [61] and the residual expression levels can be derived again after subtracting the estimated hidden factor contributions. This technique is popular, but an alternative approach is to use expression-derived PCs [62] to remove non-genetic expression variation. The significant (*p* < 0.05) PCs in the Tracy-Wisdom test [63,64] can be used as covariates during association testing or can be regressed out from the expression data. Some studies used arbitrarily either three, five, or up to ten PCs as covariates to remove hidden factors, but others (e.g., [21,22]) repeat eQTL analysis with varying numbers of PCs to identify the optimum number of PCs that maximize the number of eQTLs identified above suitable significance thresholds.

### 4.5. Expression Quantitative Trait Locus (eQTL) Analysis

eQTLs can have effects in *cis* and in *trans*. *Cis*-effects are taken when there are differences in expression levels of genes within 1 Mb of the associated SNP; *trans*-associations arise from relevant SNPs affecting expression of more distant genes, including genes on other chromosomes. MatrixEQTL [65] is currently the most popular eQTL analysis tool. It is a fast additive linear regression model for performing these tests for *cis* and *trans* associations separately. If the PCs and other covariates have not already been removed from the expression sets, then they can be used as covariates inside MatrixEQTL. Additively recoded (0,1,2) SNP data is used along with an expression matrix to perform the tests. Although there are options in MatrixEQTL to test expression against three genotypes per SNP, additive recoding detects eQTLs better. In the case of testing GWAS SNPs, it is better practice if the SNPs are additively recoded by the risk allele rather than the reference allele such that the beta coefficients provide the direction of expression regulation in relation to the risk allele. This information can be particularly useful for downstream pathway and enrichment analysis as well as to compare effect directions in different cell types. In a large meta-analysis study, Westra et al. [25,26] developed a pipeline where QTLs were determined by using Spearman rank correlation on genotype dosages in each cohort. Then, a meta-analysis was performed to combine the results by a weighted z-score method.

### 4.6. Statistical Significance and Permutation Analysis

Generally, all eQTL findings at a Benjamini-Hochberg adjustment false discovery rate (FDR) [20] under 0.001 are considered significant (as in [19]). FDR adjustment is separately applied for *cis*- and *trans*-associations. In some studies [23,24], probe-variant pairs with adjusted *p*-values less than 0.05 were deemed significant. However, this simple correction procedure is confounded by LD between SNPs tested, correlation between probes, and differences in minor allele frequencies (MAF) between the SNPs. To address these issues, permutation based strategies were introduced [25] to correct for multiple testing. For this purpose, the eQTL analyses are repeated up to 10,000 times using a R “*q*-value package” [66] with permuted sample labels and null *p*-values derived. The *q*-values are then derived over the null *p*-values, and eQTL associations with *q*-values < 0.05 are generally deemed significant.

### 4.7. Colocalization: Overlap between eQTL for a Gene and GWAS SNPs for Disease

There are a number of tools available to link the eQTL to a disease associated variant. For example, Sherlok [67] calculates a SNP-level Bayes factor using observed GWAS and eQTL *p*-values of SNPs to determine the likelihood that expression changes in the gene mediate disease risk as opposed to the gene not being related to disease. Alternatively, co-localization directly evaluates whether two associations (GWAS and eQTL) in the same locus, observed in different cohorts, were due to the same underlying effect. The R package “coloc” [68] is a well-calibrated Bayesian framework that considers spatial similarities in association data across sets of SNPs; “gwas-pw” [69] is a similar method with the addition of hierarchical priors, and it optimizes model parameters; HEIDI/SMR15 [70], applies Mendelian randomization between traits.

### 4.8. Joint eQTL Analysis for Multiple Cell Types/Tissues

In this paper, we compared the eQTLs of T1D in different immune cells by comparing the results reported in different studies. This technique has its disadvantages where there is a possibility that cell-specific eQTLs were not detected in other cells due to incomplete power. Several approaches [71,72] have been developed to perform joint eQTL analysis in multiple cells or tissues primarily using a Bayesian framework to overcome these issues. These techniques were recently applied to perform analysis on 45 tissues [73], and data has been made available in the GTEx online portal (http://www.gtexportal.org). The GTEx data can be accessed to identify the eQTLs associated with disease associated SNPs as well as their proxy (high LD) SNPs. GTEx data can be also useful in performing tissue specific enrichment analysis, whereby the most affected tissue for a given list of disease-associated SNPs can be identified by simply counting the number of eQTL instances identified per tissue.

### 4.9. Imputation of Gene Expression Profiles

PrediXcan [74] is a recently developed method aimed at saving the costs of expensive transcripome sequencing. It can impute transcriptome-wide expression profiles for Caucasian samples based on reference transcriptome datasets from large studies such as Genotype-Tissue Expression (GTEx) [73], Genetic European Variation in Health and Disease (GEUVADIS) [75], and Depression Genes and Networks (DGN) [76] where both expression and SNP data are available. In the current release of PredictDB (a database associated with the tool), the authors of PrediXscan only included genes that had a false discovery rate ≤5% (for example 11,553 autosomal genes in whole blood) based on the elastic net models used to generate the SNP weights. The quality of the transcriptome imputation depends highly on the number of SNPs included in the gene expression prediction model: the more SNPs, the better the imputation quality. Prediction models are available for many tissues to allow evaluation of eQTL associations with diseases before undertaking actual gene expression measurements.

### 4.10. Chromatin Conformation Capture and Linking GWAS SNPs to Target Genes

Chromatin conformation capture (3C) and variants of this approach (4C, 5C, Hi-C, and ChIA-PET) probe long-range interactions by utilizing formaldehyde-directed cross-linking of genomic modules that are close in physical space [77]. A recent paper [78] provided high-resolution analysis of interactions involving almost all annotated promoters (Fantom5 [43], ENCODE [42]) in 17 human primary blood cell types. Links were identified between disease-associated variants with their putative target genes by integrating chromatin-interaction with population genetics data. The data can be accessed via an online portal: www.chicp.org.

### 4.11. Pathway, Network, and Enrichment Analysis

After identification of target genes regulated by the disease variants, pathway and enrichment analysis follow to provide insights into potential biological mechanisms the genes might be involved in. These analyses can be performed on a cell-type specific basis or on combined tissues. There are several tools available to perform these analyses. DAVID v6.8 [79] is a very popular enrichment analysis tool that has been extensively applied to discover pathways for a given set of genes. Typically, annotation terms meeting Benjamini-Hochberg *p* < 0.05 (adjusted for the number of terms) are considered significant. Gene-set collections available in the Molecular Signature Databases (MSigDB) [28] of the Broad Institute can be also tested for enrichment using their web interface (http://software.broadinstitute.org/gsea/msigdb/annotate.jsp). Again an FDR adjusted *p*-value is computed for all terms for measuring significance. Both DAVID and MSigDB provide options to test Gene Ontology (GO) terminologies in addition to canonical pathways; GO is useful in identifying significant biological, cellular, and molecular functions associated with the gene lists. If the direction of eQTL associations with respect to the risk allele is known, GO analysis can be separately applied to up- and down-regulated genes so as to identify functions that are either magnified or diminished in relation to susceptibility.

Other popular enrichment tools such as GWAS Analysis of Regulatory or Functional Information Enrichment with LD correction (GARFIELD) [80] and Genomic Regulatory Elements and Gwas Overlap algorithm (GREGOR) [81] also provide cross-verification for significantly enriched terms. Ingenuity Pathway Analysis (IPA) is a commercial software for performing enrichment analysis with special options to perform toxicity analysis and identify drugs associated with candidate genes. Network analysis allows connecting target genes with related molecules and helping identify associated functions. This type of analysis can be performed with the help of the Cytoscape-GeneMania [82] module that allows for the connection of genes based on co-expression patterns. An MCODE (molecular complex detection) plugin within Cytoscape [83] allows for identifying clusters of sub-networks within highly connected networks, functions of which can be separately defined within Cytoscape itself. Such types of analysis will help to shed insights into the biological functions underlying susceptibility.

## 5. Conclusions

Type 1 Diabetes is a complex genetic disease with reports of over 60 loci that increase a person’s risk of developing the disease. Most of the risk loci do not mediate disease susceptibility via missense changes to coding regions [19]. We investigated the role of risk SNPs in affecting gene expression in various immune related cells, combining the results of expression studies conducted in B-cells, monocytes, dendritic cells, EBV-transformed B-cells, and CD4+ and CD8+ T-cells. Our results revealed that there were 90 *cis*-regulated genes and ten *trans*-regulated genes that were evidenced in multiple cells (or studies). In addition, there were 34 and 22 highly significant cell-specific *cis*- and *trans*-regulated genes, respectively. We provided a methodology for the identification of eQTLs associated with T1D SNPs in different immune cells and suggested alternative methods to improve and overcome the statistical limitations (power) of the techniques used. We have also discussed several advancements in the area of linking GWAS SNPs to functional variants using next generation sequencing techniques. Future study designs for testing T1D GWAS variants will need to incorporate these advancements and find ways to integrate data from various sources as discussed in the paper to further improve our understanding of the disease susceptibility.

## Figures and Tables

**Table 1:** List of *Trans*-regulated genes found with association in multiple cell types.

T1D SNP	Locus	Chr	Gene	B	EBV	PMA	CD4	CD8	Mono	LPS2	LPS24	IFN	Blood
rs7111341	*INS*	11p15.5	*AQP9*				6.0	5.6					
			*IP6K2*	14.2	18.2	11.9	17.8	20.7	16.9	7.7	13.3	10.0	
			*LAP3P2*	51.7			14.6	14.4	13.9				
rs11171739	*ERBB3*	12q13.2	*MAFG-AS1*		6.9	5.6	17.0	19.9					
			*MIR130A*		36.2	27.8	25.7	21.8					
			*MIR1471*		6.9	5.6	8.5	13.0					
			*DPF2*					6.8					12.0
rs3184504	*SH2B3*	12q24.12	*UBE2L6*		7.9	4.7							7.9
			*STAT1*		5.5								7.5
rs17696736	*C12Orf30*		*UBE2L6*		5.7								5.3

SNP—single nucleotide polymorphisms; B—CD19+ B cells; EBV—Epstein-Barr virus resting; PMA—Phorbol 12-myristate 13-acetate stimulated EBV; Mono—monocytes; LPS2, LPS24—lipopolysaccharide stimulated monocytes; IFN—interferon-*γ* stimulated monocytes.

**Table 2:** List of cell-type specific *trans*-regulated genes.

EBV Cells		Peripheral Blood	
SNP	*Trans*-Gene	SNP	*Trans*-Gene
rs1990760	*LOC643997*	rs1701704	*CCL5*
rs10499194	*TUBB6*	rs1701704	*CRLF3*
rs7804356	*SLC39A8*	rs2058660	*CYP2C19*
rs12251307	*DERA*	rs3184504	*FOS*
rs947474	*MEIS2*	rs3184504	*GBP4*
rs3842727	*ID2*	rs11171739	*MIF*
rs1738074	*IRF8*	rs3184504	*NALP12*
rs1265565	*NCOA7*	**CD4+**	
rs12908309	*FAHD1*	rs1265565	*ZMYM5*
rs2290400	*TEX9*	rs11711054	*GRAMD1B*
rs763361	*P2RY11*	**Monocytes**	
rs7221109	*EIF5A*	rs11171739	*KCTD11*

## Supplementary Materials

The following are available online at www.mdpi.com/2073-4425/8/6/167/s1. Table S1: List of *cis*- and *trans*-regulated gene immune cells.
